# Global research trends in surgical management of choledochal cysts: a bibliometric analysis from 2000 to 2024

**DOI:** 10.3389/fmed.2025.1611045

**Published:** 2025-07-11

**Authors:** Xingjian Liu, Guodong Zhu, Jiayi Zhang, Yulong Mu, Zongxing Qiao, Chuan Wang

**Affiliations:** ^1^Department of Pediatric Surgery, Qilu Hospital of Shandong University, Jinan, China; ^2^Department of Orthopedics, Qilu Hospital of Shandong University, Jinan, China; ^3^Department of Internal Medicine, Wudi Hospital of Traditional Chinese Medicine, Shandong, China; ^4^Department of Endocrinology and Metabolism, Qilu Hospital, Shandong University, Jinan, China; ^5^Institute of Endocrine and Metabolic Diseases of Shandong University, Jinan, China; ^6^Key Laboratory of Endocrine and Metabolic Diseases, Shandong Province Medicine and Health, Jinan, Shandong, China; ^7^Jinan Clinical Research Center for Endocrine and Metabolic Diseases, Jinan, Shandong, China

**Keywords:** choledochal cysts, surgery, bibliometric, complication, management

## Abstract

**Introduction:**

Choledochal cysts (CCs), which are congenital biliary anomalies prevalent in Asia, predominantly affect children and carry the risk of severe complications, including malignancy. Surgical intervention, evolving from open to minimally invasive (laparoscopic/robotic) techniques, remains the cornerstone of management. Despite growing research, no bibliometric analysis has mapped the global trends in CCs surgical studies. The aim of this study was to comprehensively analyze the advances in the surgical treatment of CCs since the 21st century and to predict future research directions.

**Methods:**

Using the Web of Science Core Collection (2000–2024), we analyzed 1,064 publications on CCs surgery via Bibliometrix and CiteSpace. The search terms included surgical techniques (e.g., “laparoscop*,” “Roux-en-Y”) and disease terms (e.g., “choledochal cyst*”). The data encompassed authorship, citations, institutions, and keywords.

**Results:**

Average Annual publications increased by 5.95%, peaking by 2023 (*n* = 93). China (*n* = 579) and Japan (*n* = 398) dominated the research output, yet international collaboration was limited (MCP rate <10%). High-impact studies emphasized long-term malignancy risks after Roux-en-Y anastomosis and laparoscopic superiority over open surgery. Keyword analysis revealed shifting foci: “management” (*n* = 241) and “children” (*n* = 204) were predominant, while “robotic surgery” emerged post-2010. Institutions such as Monash University and the Capital Institute of Pediatrics led productivity, with the Journal of Pediatric Surgery being the top publisher.

**Conclusion:**

Surging research on CCs surgery highlights evolving priorities: bibliometrics reveal a growing focus on long-term outcomes and minimally invasive techniques (laparoscopic/robotic). Enhanced preoperative and postoperative management and novel diagnostic methods are critical. Global collaboration and standardized training remain pivotal for optimizing patient prognosis.

## Introduction

Choledochal cysts (CCs) are congenital disorders presenting as abnormal cystic dilatation of the intrahepatic and/or extrahepatic bile ducts (1), and can develop at all ages; however, approximately 80% of CCs are diagnosed before the age of 10 years (1, 2). CCs are thought to be more common in Asia than in Europe and the Americas, with incidence rates ranging from 1:1,000 live births in Japan to 1:13,500 live births in the United States (3), and approximately four times the incidence of CCs in females than in males in Western countries (4). CCs can lead to several serious complications such as recurrent cholangitis, acute pancreatitis, secondary biliary cirrhosis, and severe malignancies (5). Surgery is considered the cornerstone of the treatment of CCs (6), and surgical resection of the lesion and reconstruction of the bile ducts are effective in reducing the long-term risk of cancer, pancreatitis, and cholangitis (7).

In the early days, CCs were treated with open surgery using Roux-en-Y hepaticojejunostomy (8). However, since the 21st century, minimally invasive surgery using laparoscopy has been increasingly used for CCs (9), and robot-assisted surgery has become a trend in pediatric surgery (10), both of which have demonstrated better intraoperative and postoperative outcomes than open surgery, and the development of surgical modalities has been effective in decreasing mortality rates in children with CCs (11, 12). Despite the fact that surgical treatment is effective in treating CCs, perioperative complications as well as complications due to poor postoperative management remain a major threat to the health of patients (13, 14). Postoperative follow-up and long-term monitoring have become necessary programs for patients with CCs after surgery (15).

Bibliometrics uses mathematical and statistical methods to quantitatively analyze information from the literature. This approach permits the examination of quantitative and distributional patterns in the literature, which helps to explore the patterns and characteristics of scientific activity in depth (16). By examining publication data (e.g., number of articles, citations, and collaborative networks), bibliometrics provides a clear view of the research landscape (17). This provides an alternative perspective to traditional analytical approaches to facilitate evidence-based decision-making in academia (18). Using this approach, we can shed light on research patterns, evolutionary trajectories, emerging hotspots, research frontiers, and future trends in specific scientific fields (19, 20).

Therefore, this study presents the first bibliometric analysis of the surgical treatment of CCs, aiming to quantitatively map the global research landscape from 2001 to 2024, to identify seminal publications and evolving research priorities, and to predict translational pathways to optimize clinical practice, which in turn will identify future international research trends in the surgical treatment of CCs.

## Methods

### Search strategy and data collection

All publications included in our study were obtained from the Web of Science (WOS), of which the Web of Science Core Collection (WoSCC) is the world’s most influential multidisciplinary indexing database of scholarly literature, consisting of 10 sub-collections of more than 12,000 prestigious scholarly journals, and provides the comprehensive data needed for bibliometric analysis, including 282 of the most influential and highest quality scientific journals relevant to our study. WoSCC provides comprehensive data for bibliometric analysis, including 282 of the most influential and high-quality scientific journals relevant to this research. The search strategy was set as follows: (TS = (“surg*” OR “laparoscop*” OR “minimally invasive” OR “Roux-en-Y” OR “hepaticojejunostomy” OR “biliary reconstruction” OR “cystic excision“)) AND TS = (“choledochal cyst*” OR ‘choledochal dilatation’ OR ‘congenital choledochal cyst’ OR ‘bile duct cyst*’). The search and screening processes are described in Supplementary Figure 1. The articles were published between January 1, 2000, and December 31, 2024. Articles and reviews have been included in the manuscript. We chose WoSCC as the sole data source because of its broad coverage, inclusion of high-impact journals, and rich citation data, which are essential for conducting high-quality bibliometric analysis. While we recognize the potential value of other databases, such as Scopus and PubMed, our focus on WoSCC was driven by its leadership position in pediatrics and surgery. The use of WoSCC ensures data consistency and comparability throughout the analysis to maintain data integrity and quality. The specific retrieval process can be seen in Supplementary Figure 1.

### Statistical analysis

Title, authors, year of publication, country/region, institution, keywords, citations, and references were obtained from the WOS database. The downloaded files are in plain-text format. Impact factors (IF) were calculated using Journal Citation Reports (JCR) 2022. For all analyses related to words, the range was selected as keyword plus, which also addressed the subject categories used for disciplinary analysis in the WOS. All data that met the inclusion criteria for this study were extracted by the researcher and exported as plain text (. txt) files. All data analyses regarding the articles were performed using the R package bibliometrix and Citespace software version 6.2. R4 (11).

## Results

### Global research trends in the surgical treatment of CCs

**Figure 1:**
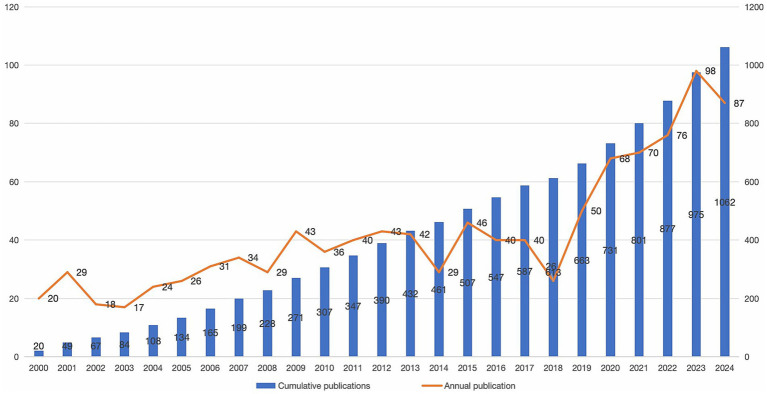
Trends in the number of articles published on choledochal cysts (CCs) surgery research. Bar graph and line graph showing changes in the number of articles published on surgery for CCs over the years.

Table 1 provides a comprehensive overview of the analysis. A total of 1,062 articles spanning a period of 24 years were retrieved from 2000 to 2024. These include 865 articles, 54 articles in proceedings papers, 142 reviews and 1 retracted review. The average annual growth rate of publications was 5.95%. The number of publications has increased from 20 in 2000 to 87 in 2024. The number of articles published each year reflects trends in the relevant research. Since 2019, there has been a rapid growth trend in the publication of articles on CCs surgery, with the highest number of publications occurring in 2023 (93) (Figure 1).

**Table 1:** General information of the included publications.

Description	Results
Main information about data	
Timespan	2000:2024
Sources (Journals, Books, etc.)	282
Documents	1,062
Annual growth rate %	6.32
Document average age	10
Average citations per doc	13.99
References	12,569
Document contents	
Keywords plus (ID)	1,062
Author’s keywords (DE)	1,563
Authors	
Authors	4,472
Authors of single-authored docs	12
Authors collaboration	
Single-authored docs	13
Co-authors per doc	5.86
International co-authorships %	8.286
Document types	
Article	865
Article; Proceedings paper	54
Review	142
Review; Retracted publication	1

### Author impact and collaboration analysis

A total of 4,472 authors contributed to the study on surgery of CCs (Table 1). The author with the highest number of publications is Li L, with 38 publications (3.57% of the total), and an H index of 17. DIAO M, YAMATAKA A and LANE GJ are also among the researchers who participated in the top ranked number of publications. Supplementary Table 1 shows the total number of papers, percentage of papers, H index, G index, and country of the top ten authors in the field of surgical research on CCs. Most of these authors were from China and Japan, with one from the UK. In terms of citations, LI L was the most cited author with 718 total citations (TC). In terms of author collaborations, Li L and Yamataka A became the most collaborating authors, but we noticed that the researchers collaborated less internationally and mainly focused on domestic collaborations (Supplementary Figure 2).

### Journal institutional contribution analysis

In total, 282 journals were included in this study. Supplementary Table 2 lists the top 10 journals by article production. JOURNAL OF PEDIATRIC SURGERY from the United States was ranked first with 99 papers, and PEDIATRIC SURGERY INTERNATIONAL and JOURNAL OF LAPAROENDOSCOPIC and ADVANCED SURGICAL TECHNIQUES were ranked second and third, with 87 and 39 papers, respectively. Among these journals, only JOURNAL OF PEDIATRIC SURGERY, SURGICAL ENDOSCOPY, AND OTHER INTERVENTIONAL TECHNIQUES, and WORLD JOURNAL OF SURGERY were ranked in Q1. Six publishers were from the USA, two from Germany, and one from Switzerland. from Italy, and one from Switzerland.

**Figure 2:**
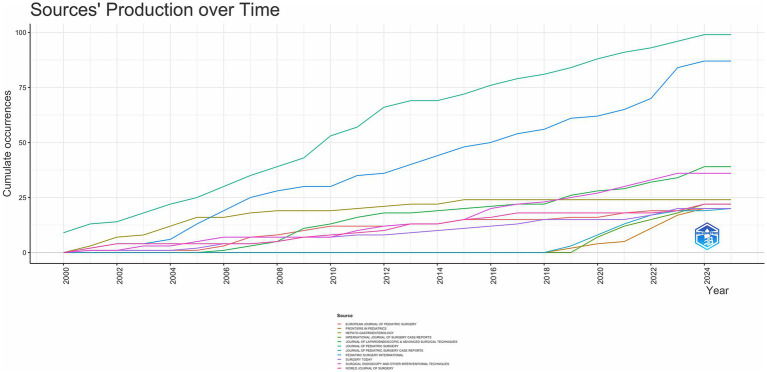
Trend of publications in top journals from 2000 to 2024.

**Figure 3:**
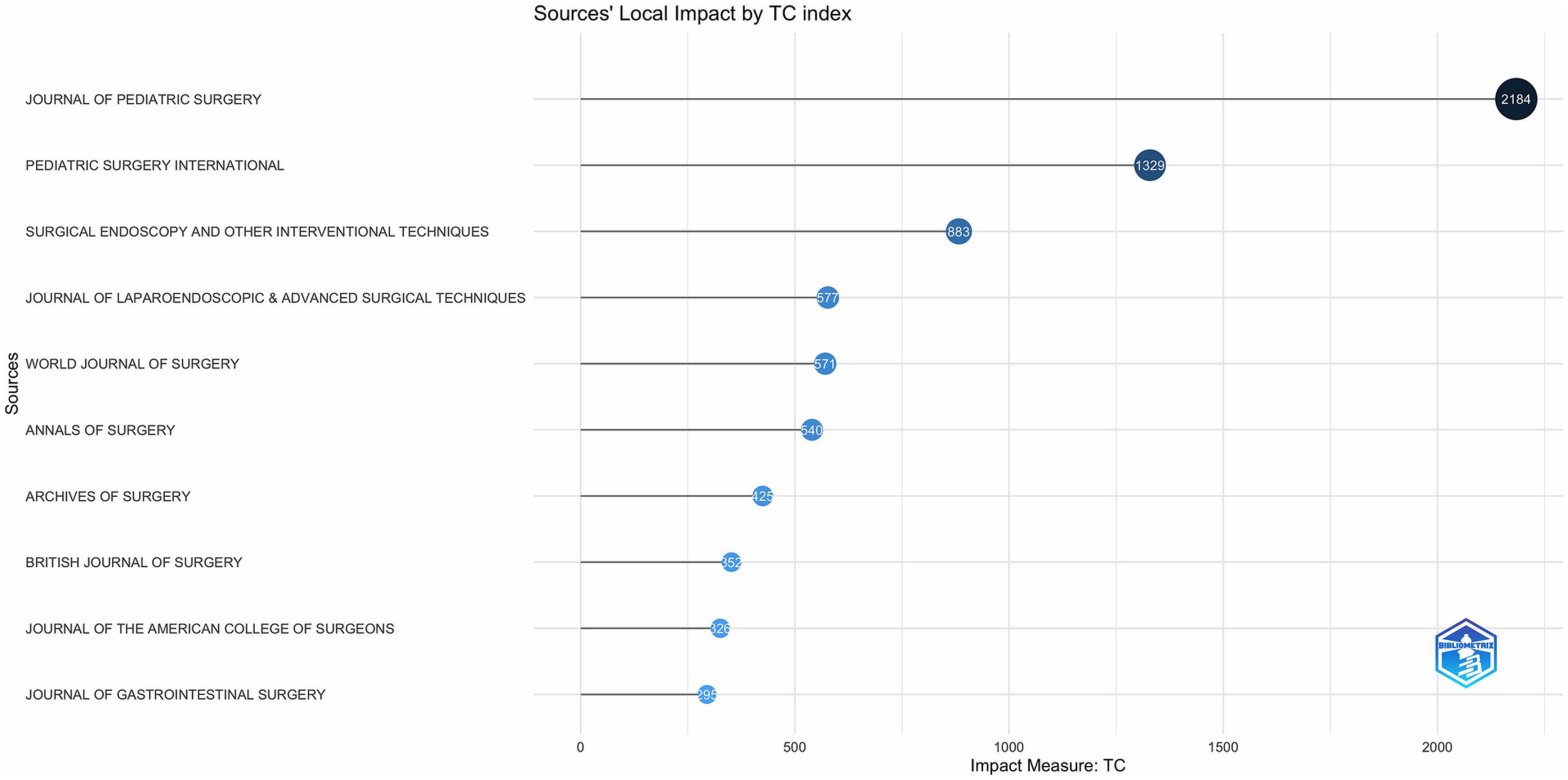
Number of publications in top journals based on TC index.

**Figure 4:**
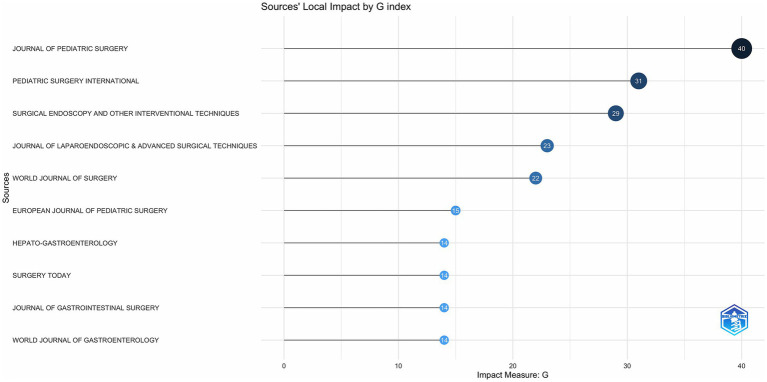
Number of publications in top journals based on G index.

**Figure 5:**
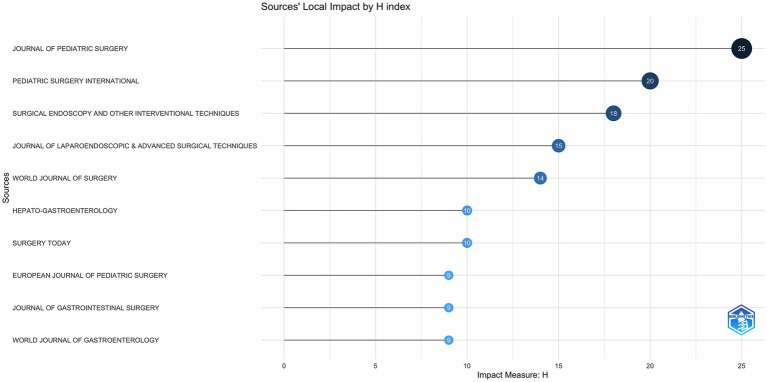
Number of publications in top journals based on H index.

As shown in Figure 2, JOURNAL OF PEDIATRIC SURGERY has led to the number of publications related to CCs surgery in the 21st century, followed by PEDIATRIC SURGERY INTERNATIONAL has been in the second place in terms of the number of annual publications since 2006, and the other journals have a much more homogeneous distribution of annual publications. In terms of TC, H Index and G Index (Figures 3–5), JOURNAL OF PEDIATRIC SURGERY, PEDIATRIC SURGERY INTERNATIONAL, SURGICAL ENDOSCOPY, AND OTHER INTERVENTIONAL TECHNIQUES are all in the top three. Were in the top three positions.

### Analysis of authors’ institutional contributions

A total of 858 author institutions were involved in research related to CCs surgery. Supplementary Figure 3 shows the top 10 institutions in terms of article output, with MONASH UNIVERSITY leading with 44 papers. Other notable institutions include the CAPITAL INSTITUTE OF PEDIATRICS (CIP), JUNTENDO UNIVERSITY, and CHONGQING MEDICAL UNIVERSITY. Supplementary Figure 3 shows the top 10 institutions in terms of article output, with MONASH UNIVERSITY leading the way with 44 articles. Supplementary Figure 4 shows the publication trend of the research institutions with the most publications over the 21st century, with Juntendo University in first place in terms of publications until 2012, and Monash University in first place in terms of cumulative publications between 2012 and 2024.

### Analysis of country/region contributions

**Figure 6:**
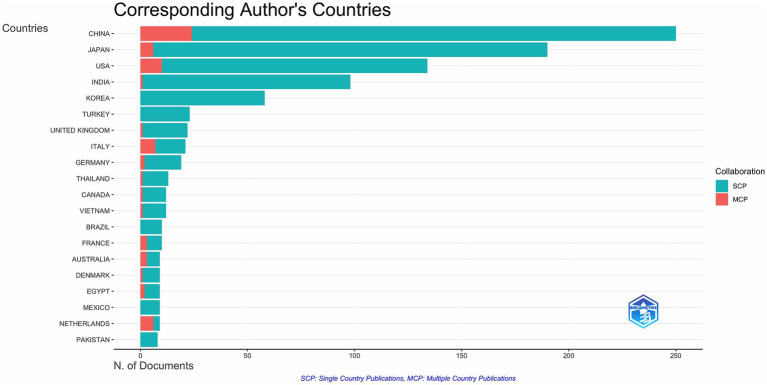
Geographic distribution of publications on the countries of the corresponding authors of global CCs surgery publications.

**Figure 7:**
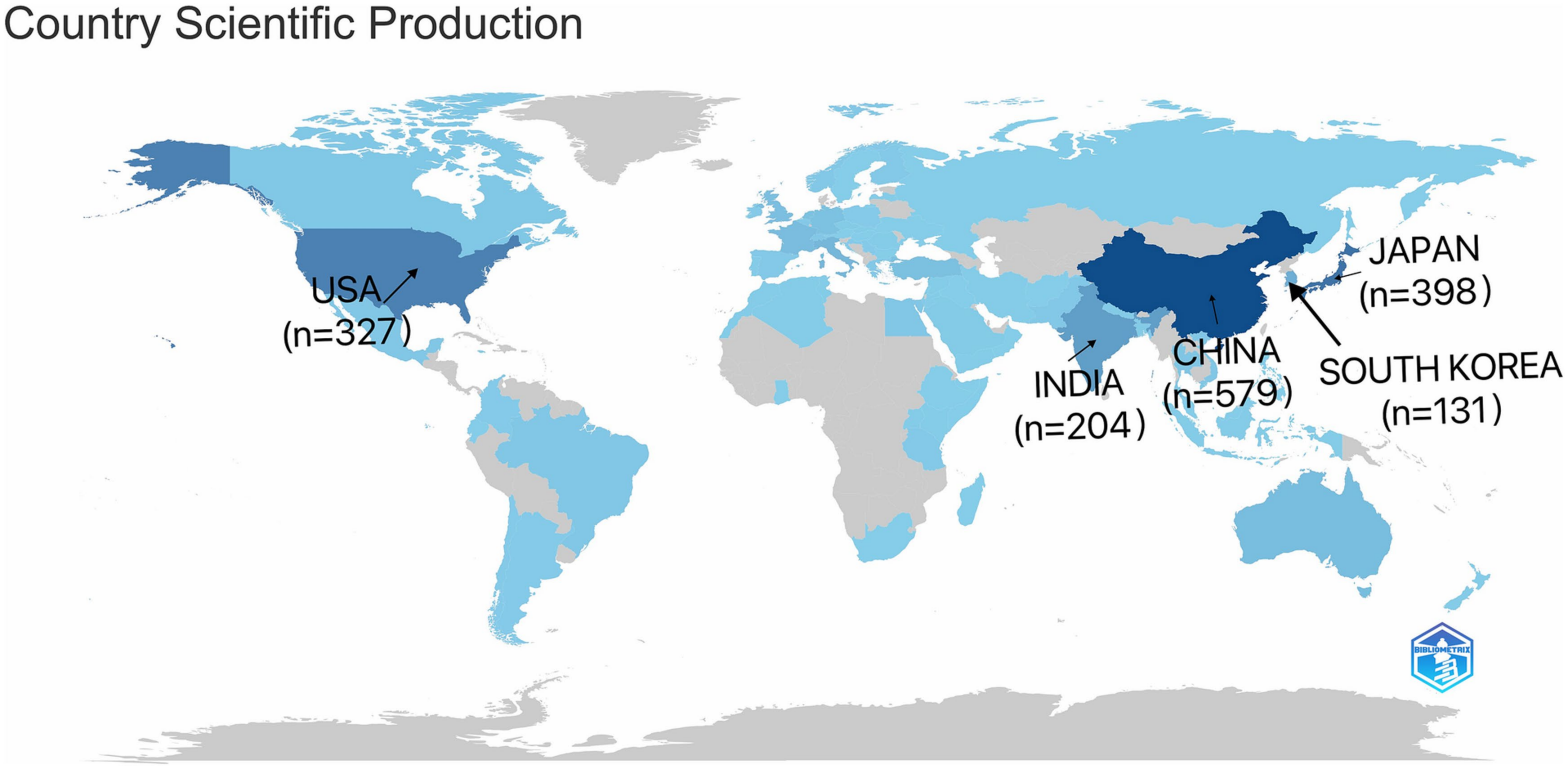
Global geographic distribution of the corresponding authors.

As shown in Figure 6, the top five participating countries were China (*n* = 579), Japan (*n* = 398), the United States (*n* = 327), India (*n* = 204), and the Republic of Korea (*n* = 131), with a combined total of 1,639 articles from these countries. Figure 7 lists the top 20 countries/regions in terms of number of participating studies, with China having the highest number of multicountry publications (MCPs) at 24. Among the top 20 countries in terms of the number of publications, the country with the highest MCP rate was NETHERLANDS (66.7%).

### Analysis of highly cited research

The most-cited publications in the field indicate the impact of research. Table 2 lists the 10 most-cited studies, all of which have been cited more than 100 times. The 2001 article “Late development of bile duct cancer in patients who had biliary-enteric drainage for benign disease: a follow-up study of more than 1,000 patients” in ANNALS OF SURGERY received the highest number of citations and demonstrated the importance of close monitoring of biliary-enteric drainage-induced chronic inflammatory changes in order to prevent late development of biliary malignancies. The second most cited article was “Comparison of endoscopic sphincterotomy and laparoscopic exploration of the common bile duct” published in the British Journal of Surgery in 2002, which demonstrated the importance of close monitoring of biliary-intestinal drainage to prevent late development of biliary malignancies. In response to choledocholithiasis, a common complication of CCs, the laparoscopic exploration of the common bile duct may be a better method of stone removal than endoscopic sphincterotomy plus laparoscopic cholecystectomy.

**Table 2:** The top 10 cited publications.

Publication	First author	Total citations	DOI
TOCCHI A, 2001, ANN SURG	TOCCHI A	165	10.1097/00000658-200108000-00011
TRANTER SE, 2002, BRIT J SURG	TRANTER SE	162	10.1046/j.1365-2168.2002.02291.x
SINGHAM J, 2009, CAN J SURG	SINGHAM J	156	NA
SOARES KC, 2014, J AM COLL SURGEONS	SOARES KC	148	10.1016/j.jamcollsurg.2014.04.023
SHETH S, 2000, AM J GASTROENTEROL	SHETH S	135	NA
CAPONCELLI E, 2008, J PEDIATR SURG	CAPONCELLI E	129	10.1016/j.jpedsurg.2007.12.058
DIP F, 2019, ANN SURG	DIP F	125	10.1097/SLA.0000000000003178
SÖREIDE K, 2004, BRIT J SURG	SÖREIDE K	119	10.1002/bjs.4815
VERKADE HJ, 2016, J HEPATOL	VERKADE HJ	117	10.1016/j.jhep.2016.04.032
FUNABIKI T, 2009, LANGENBECK ARCH SURG	FUNABIKI T	115	10.1007/s00423-008-0336-0

### Keyword analysis

As shown in Supplementary Figure 5, which illustrates the proportion of core subject terms attributed to each institution and country, the association and distribution of countries, institutions, and keywords in collaborative research on surgical treatment of CCs were elucidated. In terms of institutions, both Monash University and the CAPITAL INSTITUTE OF PEDIATRICS had a high level of understanding of “children,” “hepaticojejunostomy “and “laparoscopy” showed particular interest. In terms of countries/regions, China and Japan significantly contributed to these hotspots. In terms of “choledochal cyst,” “laparoscopy,” “children,” “hepaticojejunostomy” and “laparoscopy,” China and Japan contributed significantly to these hotspots. Hepaticojejunostomy, biliary atresia’, ‘pancreaticobiliary maljunction, “congenital biliary dilatation,” cholangiocarcinoma,’ ‘choledochal cysts,’ palliative care Of the 10 keywords “children,” “congenital biliary dilatation,” “cholangiocarcinoma,” “choledochal cysts” and “pediatric,” China showed a high interest in “children,” while Japan emphasized on “congenital biliary dilatation.” Congenital biliary dilatation.”

**Figure 8:**
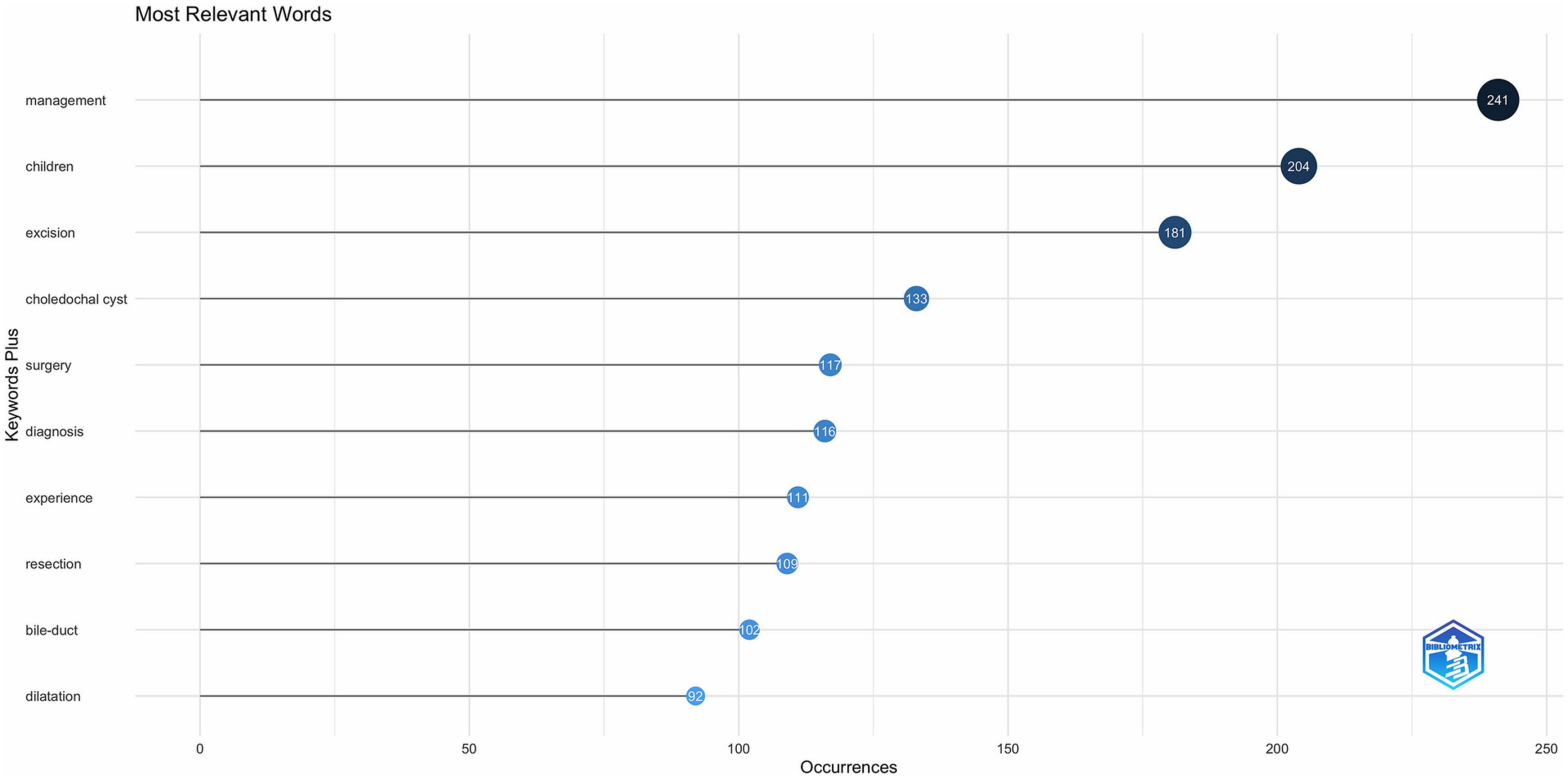
Top ten keyword clouds in the field of CCs surgery.

**Figure 9:**
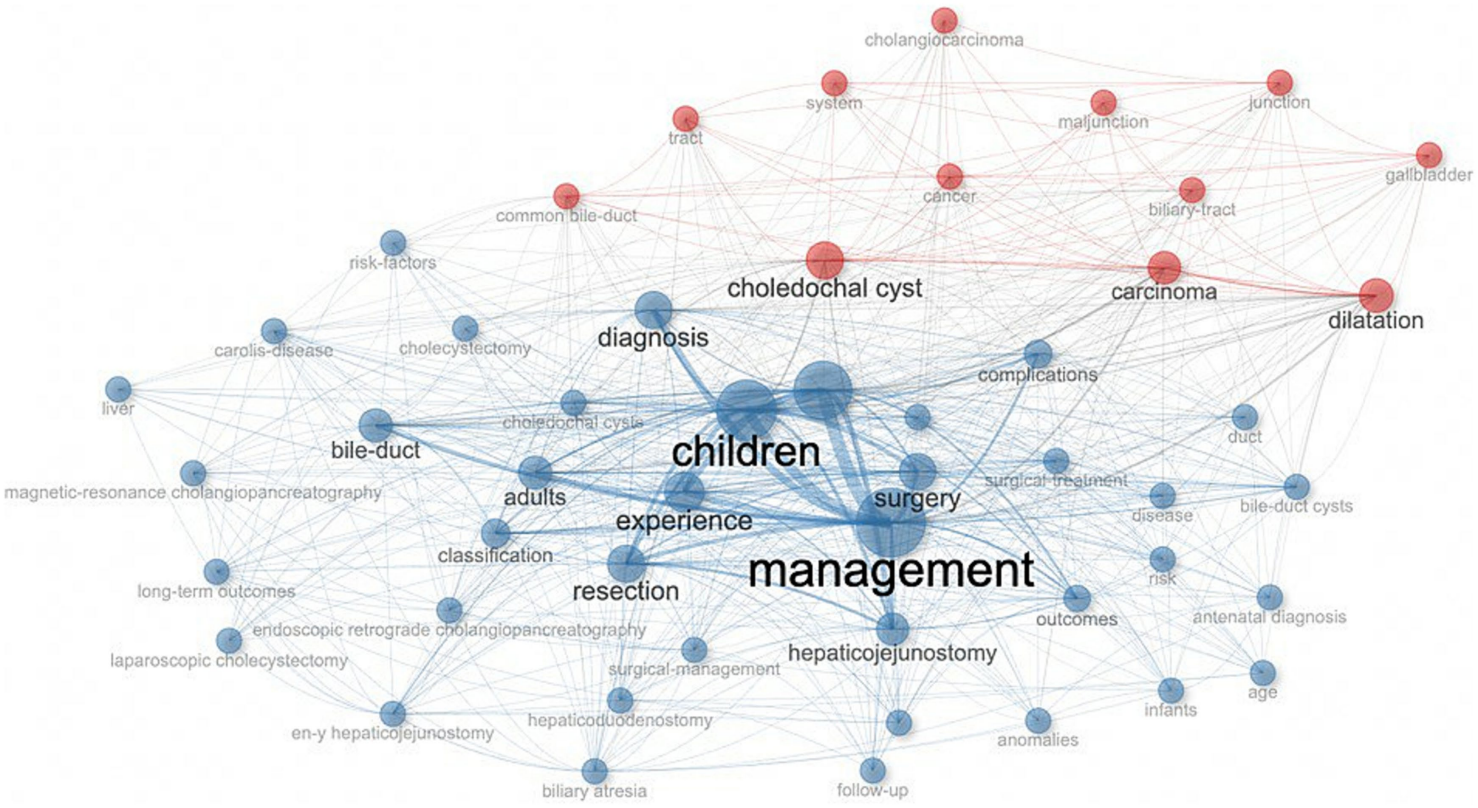
Keyword co-occurrence map in the field of CCs surgery.

Figures 8, 9 show the top 10 keywords and the connections between them. The top 10 keywords include: management, children, excision, choledochal cyst, surgery, diagnosis, experience, resection, bile-duct, dilatation, and the most frequently used keywords are “management” (*n* = 241) and ‘children’ (*n* = 204). From these keywords and their associations, we inferred that the primary study population was children and that the main focus of the study was on preoperative diagnosis and the choice of procedure and management of CCs after surgery.

**Figure 10:**
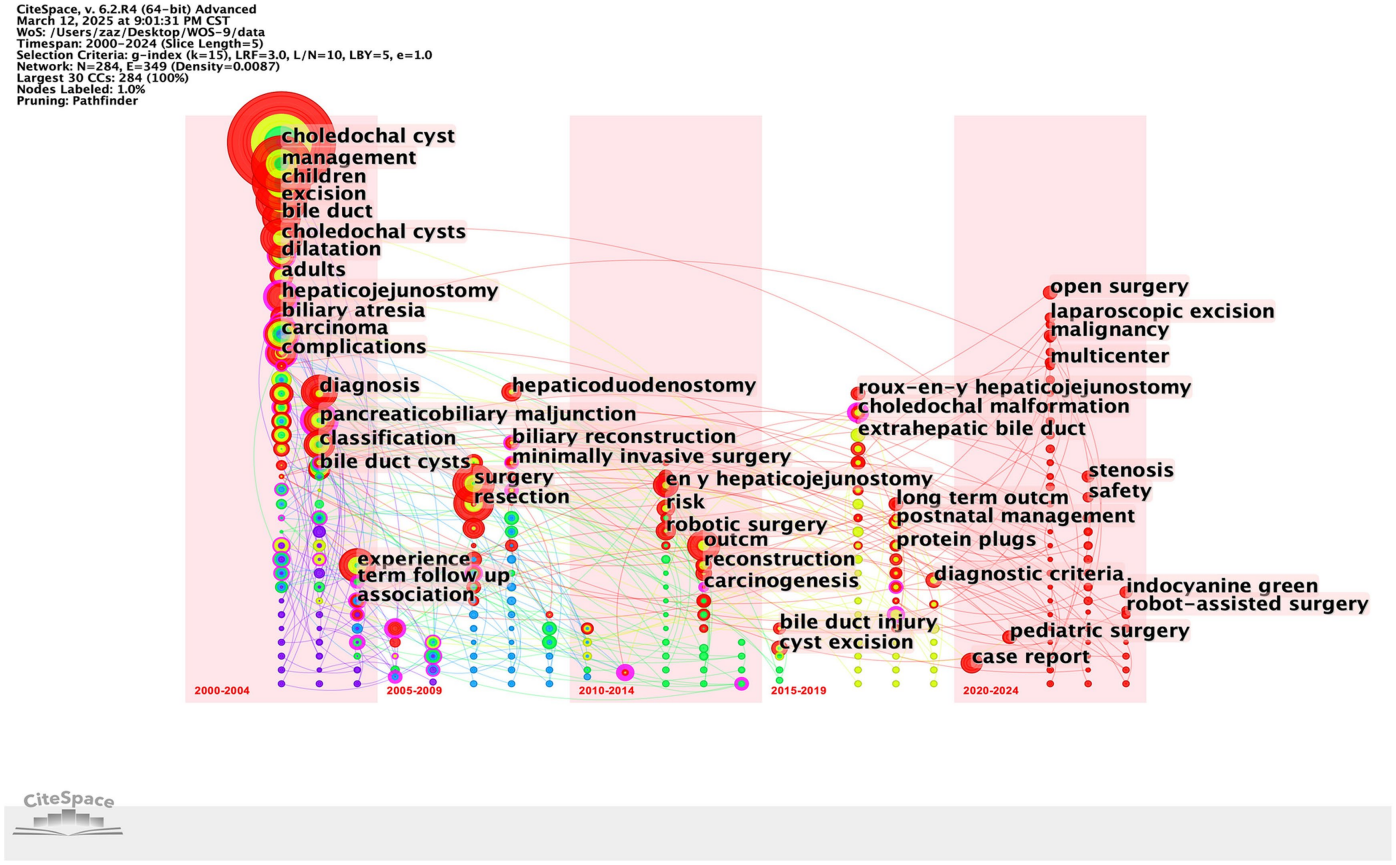
Thematic evolution of research in the field of CCs surgery in the 21st century.

**Figure 11:**
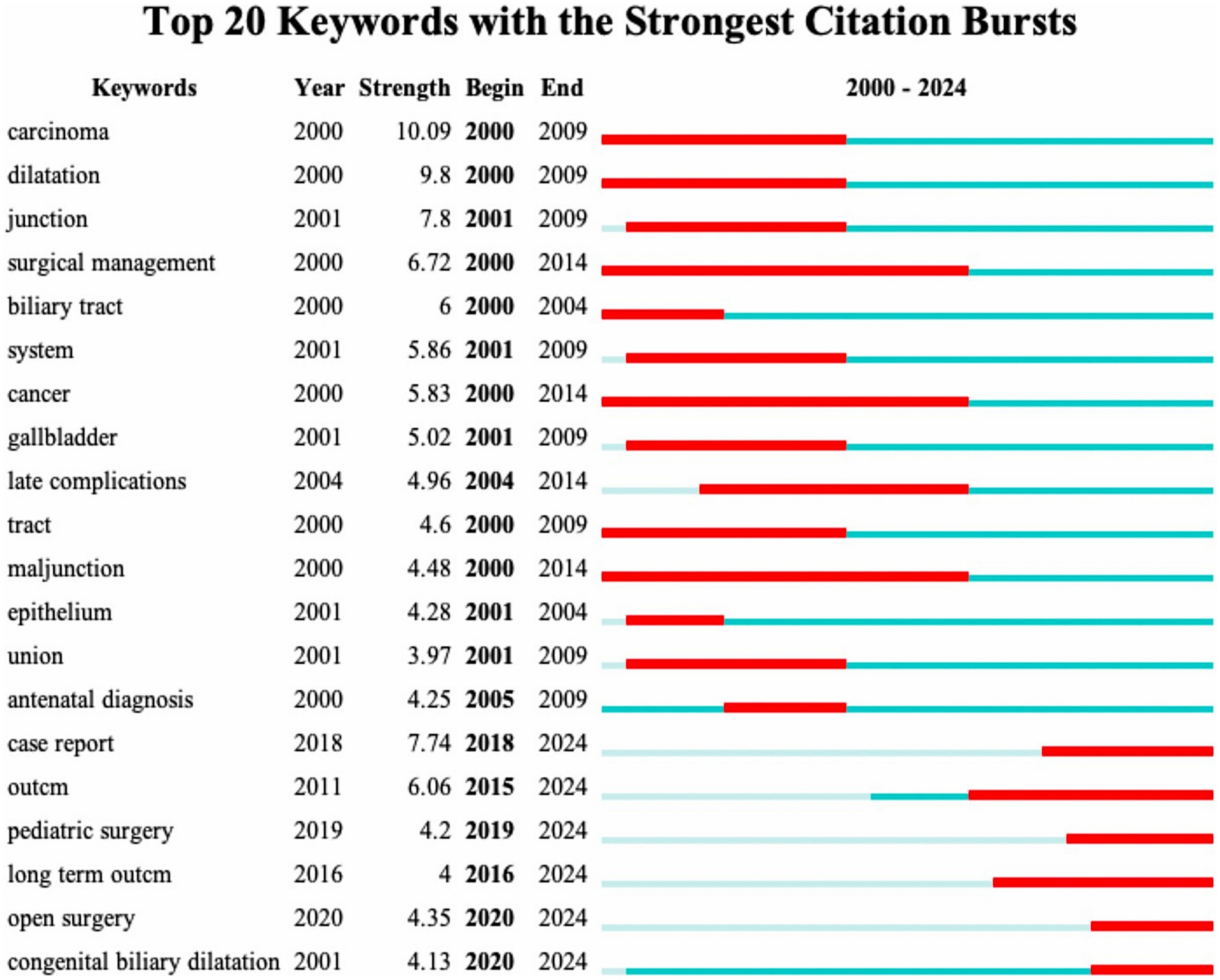
Keyword emergence map in the field of CCs surgery in the 21st century.

Figures 10, 11 show the thematic evolution and prominence in the 21st century, with the words cancer, junction, and late complications evidencing the early focus of research centered on complications and pathologic changes in the disease itself. Prior to 2010, empirically based diagnosis of CCs and management of postoperative complications of CCs became the focus of research, with the words complications, management, diagnosis, and experience appearing more frequently. After 2010, novel surgical approaches, such as minimally invasive surgery, robotic surgery, and laparoscopic excision, began to dominate the research. From around 2018, words such as postnatal management and case reports represent the surgical management of CCs into periods such as early diagnosis and individualized protocol treatment. The continued appearance of words, such as long-term outcomes from 2016, proves that the management of long-term postoperative outcomes in patients with CCs has become a hot topic at present. Notably, the increased frequency of open surgery in 2020 may signify the completion of several early multicenter prospective cohort studies comparing the efficacy of surgical procedures for CCs.

## Discussion

Since the beginning of the 21st century, the interest of researchers in the surgical treatment of CCs has continued to increase. In particular, since the beginning of 2019, there has been a significant increase in research results in this field, which may be attributed to the rise in interest in cholestatic diseases in children, of which CCs are an important component (21). Consequently, research organizations have increased their support for research on CCs, and research funding has been increasing, contributing to the rapid development of this field.

The prevalence of CCs in Asia is among the highest in the world, and China and Japan represent this (22–24). This explains why China and Japan have participated in the largest number of studies related to the surgical treatment of CCs, and four of the top five countries in terms of the number of participating studies are located in Asia. The most involved author was LI L (Long Li) from the Capital Institute of Pediatrics, China, whose research focused mainly on laparoscopic and robotic surgery for CCs and its complications (11, 25, 26), exploring the advantages of laparoscopic and robotic use for CCs surgery, especially in total cyst excision of choledochal cysts and Roux-en-Y hepatoenterostomy (27, 28). These studies provide strong evidence and support for the use of laparoscopic and robotic surgery for CCs. Also the country with the highest number of MCPs. However, we should also note that the top five countries have low MCP rates and that inter-author collaborations are basically within the country, which illustrates the limited international collaborations in CCs and the potential to improve research competitiveness through cross-national collaborations, highlighting the need for broader international inter-institutional collaborations. The increased risk of cholangiocarcinoma after Roux-en-Y anastomosis requires multinational cohorts to establish evidence-based surveillance programs (29). Differences in robotic surgery adoption rates between high-and low-income regions call for increased international collaboration in sharing technical training frameworks for global public health building. Regional genetic differences in the pathogenesis of CCs call for studies in diverse populations to develop precision prevention strategies.

Highly cited publications provide valuable insights into influential research. The most cited article published in ANNALS OF SURGERY “Late development of bile duct cancer in patients who had biliary-enteric drainage for benign disease: a follow-up study of more than 1,000 patients” demonstrated a link between biliary-enteric anastomosis and the development of biliary malignancies and revealed that the Roux-en-Y technique is expected to produce a lower incidence of cholangitis (29). These studies emphasize the need for proper surgical selection for the management of postoperative complications. The second most cited article titled Comparison of endoscopic sphincterotomy and laparoscopic exploration of the common bile duct compared endoscopic sphincterotomy and laparoscopic exploration of the common bile duct (29).

and laparoscopic exploration for bile duct stone removal, demonstrating the advantages of laparoscopic surgery in terms of reducing complications and providing support for the early use of laparoscopy in biliary surgery (30). The use of novel surgical approaches is necessary in the surgical treatment of CCs, while surgeons should not ignore the presence of complications when dealing with patients with CCs and should individualize the diagnosis and treatment for the conditions present in different patients in order to reduce postoperative recurrence and complications.

The keyword analysis highlights the main research trends in the surgical management of CCs. The most frequent word “management” proves that CCs surgical research in the 21st century has focused on preoperative and postoperative course management. The most frequent words “resection,” “excision” and “surgery” illustrate that the surgical resection protocol for CCs represented by the Roux-en-Y hepaticojejunostomy for cyst removal is the most popular one in the 21st century. Roux-en-Y hepaticojejunostomy for cysts is the primary surgical resection option for CCs. Therefore, the management of complications after resection of CCs is an important area of research, and the development of biliary tract cancer after resection of CCs is mainly observed in patients with type I and type IV cysts (31). Although the surgical removal of CCs reduces the risk of developing biliary tract cancer (32), the risk of malignancy does not return to baseline population levels (33). Secondary postoperative cholangitis and bacterial infection are risk factors for poor prognosis and even cancer development (34). Therefore, the prevention of infection and intraoperative attention to pancreaticobiliary injuries are important aspects of management (35). The high frequency of the word “children” indicates that although CCs are diagnosed in all age groups, surgery in childhood is still predominantly performed (36). The term “diagnosis” illustrates the great significance of preoperative diagnosis, and early differentiation of CCs from other biliary disorders such as biliary atresia by prenatal ultrasound and MMP7 monitoring is the focus of clinical research (21). “Dilatation,” on the other hand, is indicative of the attention paid to the morphological aspects of pathological changes in CCs, and the different pathologic and structural alterations caused by the numerous subtypes of CCs pose a challenge for definitive diagnosis and surgical procedures to be performed (37, 38). The evolution of keywords over time also shows that the research on CCs in the 21st century has developed a “structure-surgery-management” model. The focus of researchers is no longer limited to the disease and the procedure itself but is more focused on the long-term prognosis of the patient, reducing the cost of patient care and readmission rates (39).

The most common surgical approaches for CCs are cystectomy and Roux-en-Y hepaticojejunostomy, particularly for type I CCs. The traditional approach is open surgery, which is associated with complications such as high trauma, intraoperative bleeding, and pancreaticobiliary injury (40, 41). Farello et al. were the first to describe cystectomy and Roux-en-Y hepaticojejunostomy using laparoscopy in a 6-year-old child with type I choledochal cyst in 1995 (42). This has marked the official use of laparoscopy for CCs. Compared with conventional open surgery, laparoscopic resection is associated with less surgical blood loss, shorter recovery time for bowel function, shorter time to tolerate diet, shorter hospitalization, and shorter time to require parenteral nutrition (43). Crucially, laparoscopy significantly reduces short-term postoperative complications of CCs and long-term complications, including strictures, cholangitis, cirrhosis, and malignancy, most notably type III CCs (11, 44). However, previous analyses have shown that laparoscopic resection is more technically demanding and surgeon experience is critical for good outcomes in pediatric laparoscopic biliary surgery (45, 46). Meanwhile, studies on long-term complications after laparoscopic surgery still remain single-center studies (47), so the management of long-term complications and comparative studies of long-term complications between novel surgical approaches and open surgery, especially large multicenter studies, remain the focus of future surgical research in CCs.

With the development of minimally invasive technologies, robot-assisted surgery, represented by the da Vinci robotic surgical system, has enabled surgeons to perform a wide range of procedures (48). The first case of robotic surgery in a child was reported in 2001 (49). After the first da Vinci robotic surgical system-assisted excision of CCs was performed in the United States by Woo et al. in 2006, robot-assisted excision of CCs has been reported worldwide (50, 51). The population undergoing surgery has also gradually expanded from older children and adolescents to neonates (10). Current studies have demonstrated that robotic-assisted CCs surgery significantly shortens the duration of the procedure, and at the same time, due to visibility and simplification in terms of maneuvering, it will be easier for the surgeon to complete the operation and improve the surgical conversion rate (12, 45). Previous studies have shown that robotic Roux-en-Y gastric bypass surgery has a significantly lower incidence of anastomotic stricture than laparoscopic surgery (52), and is also superior to laparoscopic surgery in terms of early complications and short-term outcomes of choledochal cyst resection and hepaticojejunal anastomosis in children (53). However, robotic-assisted surgery also faces the problems of higher surgical costs, high maintenance costs, and unfamiliarity of beginners with the strength of the robotic arm system, which may lead to poor outcomes, such as implant breakage (45). Strong clinical training and education in robot-assisted surgery, as well as reducing the cost of robot-assisted surgery through upgrading, is the future of pediatric surgery (10). Meanwhile, similar to laparoscopic surgery, there are still insufficient studies on the long-term postoperative outcomes of robot-assisted CCs surgery, which will be the direction of robot-assisted CCs surgery research in the future.

Preoperative diagnosis is a key aspect of CC surgery. Owing to the non-specificity of the clinical manifestations of CCs, imaging tools have been used as the main methods to diagnose CCs (54). Early endoscopic retrograde cholangiopancreatography (ERCP) is considered the most accurate diagnostic tool to characterize the different types of choledochal cysts and to detect associated ductal anomalies (55). In 2005, Do Hyun Park et al. proposed that magnetic resonance cholangiopancreatography (MRCP), instead of ERCP, would become the gold standard for the diagnosis of CCs, which can describe the anatomical structure of cysts in detail with–90-100% sensitivity and 90% specificity (56). It can characterize the anatomical structure of cysts in detail with 90–100% sensitivity and 90% specificity, thus determining the staging of CCs to specify the type of operation (56). With the rapid development of AI technology, AI-enabled quantitative MRCP (MRCP+) has emerged as a new diagnostic tool for biliary diseases (57). MRCP+ can process 3D MRCP acquisitions with AI-driven pathfinding algorithms and renal tubule enhancement to derive quantitative parametric models of the biliary tree and pancreatic ducts (58), which provides quantitative surgical indications for biliary diseases. The application of MRCP+ to the preoperative diagnosis of CCs will change the subjective nature of the current surgical indications for CCs. The combination of multi-omics analysis techniques with artificial intelligence technology will help in the development and refinement of surgical plans (59). Prenatal diagnosis is also an important part of preoperative management of CCs. Diao et al. reported in their randomized controlled trial that patients with prenatal diagnosis and early surgical resection of the cysts had a lower risk of liver fibrosis over time (60). Aggressive prenatal diagnosis for early detection of the disease would positively impact the preoperative management of CCs, as well as surgical outcomes.

In terms of long-term prognosis after surgery, annual repeat ultrasound and Ca19.9 are recommended for lifelong follow-up because of the risk of anastomotic stenosis, cholangitis, intrahepatic stone formation, and a higher risk of malignant cancers after resection of CCs than in the normal population (54). The classification of CCs into different subtypes, whether open or laparoscopic, should be enhanced, and the Todani classification is essential to guide treatment (61). Implementing precise and individualized prognostic protocols for different individual patients with different intraoperative conditions is a trend in prognostic management (2).

This analysis provides actionable insights for optimizing the treatment of choledochal cysts. Given the advantages of laparoscopic total cystectomy in reducing perioperative morbidity and long-term complications, surgeons should consider it as the treatment of choice for type I/IV cysts, especially in the pediatric population (11, 43, 44). Robotic-assisted techniques should be considered for complex reconstructions or neonatal cases when institutional resources allow, and cost constraints should be addressed through structured training programs while recognizing their technical advantages (10, 12, 45, 53). Preoperative evaluation must include MRCP as a diagnostic cornerstone for anatomical characterization and Todani classification (56), and prenatal diagnosis requires early postnatal intervention to reduce the risk of liver fibrosis (21, 25, 60). Postoperative protocols require lifelong surveillance through annual ultrasound and CA19-9 monitoring to reduce the likelihood of malignancy (33, 54), as well as meticulous intraoperative techniques to prevent pancreaticobiliary duct injury and infection (34, 35). Finally, the establishment of multinational registries to track longitudinal outcomes of minimally invasive approaches would close current evidence gaps and standardize management paradigms (47).

This study is the first to use bibliometric methods to investigate the development and prospects of surgery for CCs. However, this study had some methodological limitations. First, reliance on the Web of Science Core Collection as the sole source of data may create a systematic bias in mapping the research landscape, as it excludes publications from emerging journals and regional databases. Second, assessment frameworks centered on citation frequency and publication volume may not adequately reflect scholarly innovation because of journal prestige and disciplinary publishing preferences. In addition, inherent limitations of the bibliometric approach remain, including the inability to contextualize citation motivations, the reduced visibility of non-English literature, and unaddressed self-citation bias. Finally, while the analysis uses the most recent available data, the rapid development of driving technologies in the AI era requires continuous data update mechanisms and the integration of technology foresight methods to address time constraints and capture dynamically evolving research frontiers and application scenarios.

## Conclusion

The bibliometric analysis suggests that surgical research on CCs is accelerating due to increasing clinical interest in long-term outcomes and new technologies (laparoscopic/robotic). Immediate priorities include improving preoperative diagnosis and postoperative complication management. These evolving trends provide strategic direction for researchers and clinicians to optimize prognostic pathways.

## Data Availability

The original contributions presented in the study are included in the article/Supplementary material, further inquiries can be directed to the corresponding author/s.

## References

[1] Brown ZJ Baghdadi A Kamel I Labiner HE Hewitt DB Pawlik TM. Diagnosis and management of choledochal cysts. HPB (Oxford). (2023) 25:14–25. doi: 10.1016/j.hpb.2022.09.010, PMID: 36257874

[2] Kong J Xia Q Xu L Jin D Sun W. Case report: Cancer-free survival after chemotherapy, targeted immunotherapy combination with proton therapy following space making technique in a patient with cholangiocarcinoma after choledochal cyst resection. Front Immunol. (2025) 15:1520248. doi: 10.3389/fimmu.2024.1520248, PMID: 39845966 PMC11750801

[3] Eiamkulbutr S Tubjareon C Sanpavat A Phewplung T Srisan N Sintusek P. Diseases of bile duct in children. World J Gastroenterol. (2024) 30:1043–72. doi: 10.3748/wjg.v30.i9.1043, PMID: 38577180 PMC10989494

[4] Soares KC Kim Y Spolverato G Maithel S Bauer TW Marques H. Presentation and clinical outcomes of choledochal cysts in children and adults: a multi-institutional analysis. JAMA Surg. (2015) 150:577–84. doi: 10.1001/jamasurg.2015.0226, PMID: 25923827

[5] Bloomfield GC Nigam A Calvo IG Dorris CS Fishbein TM Radkani P. Characteristics and malignancy rates of adult patients diagnosed with choledochal cyst in the west: a systematic review. J Gastrointest Surg. (2024) 28:77–87. doi: 10.1016/j.gassur.2023.11.007, PMID: 38353080

[6] Ho IG Ihn K Jeon HJ Lee DE Han SJ. Optimal timing of surgery for prenatally diagnosed choledochal cysts. Front Pediatr. (2023) 11:1308667. doi: 10.3389/fped.2023.1308667, PMID: 38078316 PMC10704026

[7] Xia HT Yang T Liu Y Liang B Wang J Dong JH. Proper bile duct flow, rather than radical excision, is the most critical factor determining treatment outcomes of bile duct cysts. BMC Gastroenterol. (2018) 18:129. doi: 10.1186/s12876-018-0862-3, PMID: 30139348 PMC6107957

[8] Moris D Papalampros A Vailas M Petrou A Kontos M Felekouras E. The hepaticojejunostomy technique with intra-anastomotic stent in biliary diseases and its evolution throughout the years: a technical analysis. Gastroenterol Res Pract. (2016) 2016:3692096. doi: 10.1155/2016/3692096, PMID: 27190504 PMC4846744

[9] Howell TC Beckhorn CB Antiel RM Fitzgerald TN Rice HE Mavis A. Contemporary trends in choledochal cyst excision: an analysis of the pediatric national surgical quality improvement program. World J Surg. (2024) 48:967–77. doi: 10.1002/wjs.12128, PMID: 38491818

[10] Jin Y Cai D Zhang S Luo W Zhang Y Huang Z. Robot-assisted abdominal surgery in children less than 5 months of age: retrospective cohort study. Int J Surg. (2024) 110:859–63. doi: 10.1097/JS9.0000000000000867, PMID: 37995094 PMC10871584

[11] Sun R Zhao N Zhao K Su Z Zhang Y Diao M. Comparison of efficacy and safety of laparoscopic excision and open operation in children with choledochal cysts: a systematic review and update meta-analysis. PLoS One. (2020) 15:e0239857. doi: 10.1371/journal.pone.0239857, PMID: 32986787 PMC7521726

[12] Li X Su Y Tian H Lu T Gong S Miao C. Clinical efficacy and safety of robot assisted surgery for choledochal cysts excisions: a systematic review and meta-analysis. Expert Rev Gastroenterol Hepatol. (2022) 16:787–96. doi: 10.1080/17474124.2022.2109464, PMID: 35939040

[13] Ronnekleiv-Kelly SM Soares KC Ejaz A Pawlik TM. Management of choledochal cysts. Curr Opin Gastroenterol. (2016) 32:225–31. doi: 10.1097/MOG.0000000000000256, PMID: 26885950

[14] Yang D Li L Diao M Xie X Ming A Tian Y. Prenatal diagnosis at different gestational times and clinical features of choledochal cysts: a single tertiary center report. Pediatr Surg Int. (2023) 39:105. doi: 10.1007/s00383-023-05374-5, PMID: 36752901

[15] Ghotbi J Yaqub S Søreide K. Management of extrahepatic bile duct cysts. Br J Surg. (2023) 110:1252–5. doi: 10.1093/bjs/znad087, PMID: 37079736 PMC10480039

[16] Pan D Wang J Wang H Wu S Guo J Guo L. Mapping the blueprint of artificial blood vessels research: a bibliometric analysis. Int J Surg. (2025) 111:1014–31. doi: 10.1097/JS9.0000000000001877, PMID: 38913439 PMC11745618

[17] Zhang L Zheng H Jiang ST Liu YG Zhang T Zhang JW. Worldwide research trends on tumor burden and immunotherapy: a bibliometric analysis. Int J Surg. (2024) 110:1699–710. doi: 10.1097/JS9.0000000000001022, PMID: 38181123 PMC10942200

[18] Çoşkun N Metin M. Scientific evolution from the definition of Hirschsprung disease to the present: a bibliometric analysis (1980-2023). Pediatr Res. (2025). doi: 10.1038/s41390-025-03927-z, PMID: 39979585

[19] Ahmad P Slots J. A bibliometric analysis of periodontology. Periodontol. (2021) 85:237–40. doi: 10.1111/prd.12376, PMID: 33226679

[20] Tan Z He Q Feng S. The collision of ChatGPT and traditional medicine: a perspective from bibliometric analysis. Int J Surg. (2023) 109:3713–4. doi: 10.1097/JS9.0000000000000662, PMID: 37566908 PMC10651228

[21] Verkade HJ Bezerra JA Davenport M Schreiber RA Mieli-Vergani G Hulscher JB. Biliary atresia and other cholestatic childhood diseases: advances and future challenges. J Hepatol. (2016) 65:631–42. doi: 10.1016/j.jhep.2016.04.032, PMID: 27164551

[22] Tannuri ACA Hara LAA Paganoti GF Andrade WC Tannuri U. Choledochal cysts in children: how to diagnose and operate on. Clin (Sao Paulo). (2020) 75:e1539. doi: 10.6061/clinics/2020/e1539, PMID: 32215454 PMC7074585

[23] Tainaka T Shirota C Sumida W Yokota K Makita S Amano H. Laparoscopic definitive surgery for choledochal cyst is performed safely and effectively in infants. J Minim Access Surg. (2022) 18:372–7. doi: 10.4103/jmas.JMAS_98_21, PMID: 35708382 PMC9306120

[24] Ishibashi H Shimada M Kamisawa T Fujii H Hamada Y Kubota M. Japanese clinical practice guidelines for congenital biliary dilatation. J Hepatobiliary Pancreat Sci. (2017) 24:1–16. doi: 10.1002/jhbp.415, PMID: 28111910

[25] Diao M Li L Cheng W. Timing of choledochal cyst perforation. Hepatology. (2020) 71:753–6. doi: 10.1002/hep.30902, PMID: 31461783

[26] Diao M Li L Li Q Ye M Cheng W. Challenges and strategies for single-incision laparoscopic roux-en-Y hepaticojejunostomy in managing giant choledochal cysts. Int J Surg. (2014) 12:412–7. doi: 10.1016/j.ijsu.2014.03.007, PMID: 24657348

[27] Diao M Li L Cheng W. Cysto-cholecystostomy: a more physiological procedure for hepatic cysts with biliary communications and cystic dilatations of main intrahepatic ducts. World J Surg. (2018) 42:2599–605. doi: 10.1007/s00268-018-4491-3, PMID: 29372374

[28] Jones RE Zagory JA Clark RA Pandya SR. A narrative review of the modern surgical management of pediatric choledochal cysts. Transl Gastroenterol Hepatol. (2021) 6:37. doi: 10.21037/tgh-20-235, PMID: 34423158 PMC8343510

[29] Weng H Fan QQ Gu J Weng MZ Zhang WJ Xu LM. Efficacy and long-term outcomes of single-balloon enteroscopy-assisted treatment for biliary obstruction after choledochojejunostomy. Surg Endosc. (2024) 38:6282–93. doi: 10.1007/s00464-024-11096-z, PMID: 39168861 PMC11525256

[30] Xu B Wang YX Qiu YX Meng HB Gong J Sun W. Risk factors and consequences of conversion to open surgery in laparoscopic common bile duct exploration. Surg Endosc. (2018) 32:4990–8. doi: 10.1007/s00464-018-6263-4, PMID: 29987563

[31] Moslim MA Takahashi H Seifarth FG Walsh RM Morris-Stiff G. Choledochal cyst disease in a Western center: a 30-year experience. J Gastrointest Surg. (2016) 20:1453–63. doi: 10.1007/s11605-016-3181-4, PMID: 27260526

[32] Mukai M Kaji T Masuya R Yamada K Sugita K Moriguchi T. Long-term outcomes of surgery for choledochal cysts: a single-institution study focusing on follow-up and late complications. Surg Today. (2018) 48:835–40. doi: 10.1007/s00595-018-1660-9, PMID: 29679145

[33] Choi JU Hwang S Chung YK. Management of intractable pancreatic leak from iatrogenic pancreatic duct injury following resection of choledochal cyst in an adult patient. Ann Hepatobiliary Pancreat Surg. (2020) 24:228–33. doi: 10.14701/ahbps.2020.24.2.228, PMID: 32457272 PMC7271105

[34] Kim HJ Kim JS Joo MK Lee BJ Kim JH Yeon JE. Hepatolithiasis and intrahepatic cholangiocarcinoma: a review. World J Gastroenterol. (2015) 21:13418–31. doi: 10.3748/wjg.v21.i48.13418, PMID: 26730152 PMC4690170

[35] Ikegame K Takano A Watanabe H Yamamoto A Miyasaka Y Furuya K. Biliary cancer developed after the reparative surgery for congenital choledochal cyst: a case report and review of the literature. Int Cancer Conf J. (2016) 6:43–9. doi: 10.1007/s13691-016-0270-x, PMID: 31149469 PMC6498380

[36] Sun Y Wang Z Liu Q. Adult type II choledochal cyst. Am J Gastroenterol. (2024) 119:1011. doi: 10.14309/ajg.0000000000002686, PMID: 38299615 PMC11142645

[37] Liu W Yin T Chen X Diao M Li L. Single-incision laparoscopic hepaticojejunostomy with selective ductoplasty for type IV-A choledochal cysts in children: a retrospective study. BMC Surg. (2024) 24:359. doi: 10.1186/s12893-024-02648-0, PMID: 39548424 PMC11566446

[38] Tao M Wang X Han J Cao L Li J Zheng S. A new classification and laparoscopic treatment of extrahepatic choledochal cyst. Clin Res Hepatol Gastroenterol. (2024) 48:102413. doi: 10.1016/j.clinre.2024.102413, PMID: 38960124

[39] Tan Y Shen Y Li L Yu J. Protocol for enhanced recovery after surgery with 3D laparoscopic excision for choledochal cysts can benefit the recovery process. Pediatr Surg Int. (2020) 36:643–8. doi: 10.1007/s00383-020-04644-w, PMID: 32219559

[40] Ryu HS Lee JY Kim DY Kim SC Namgoong JM. Minimally-invasive neonatal surgery: laparoscopic excision of choledochal cysts in neonates. Ann Surg Treat Res. (2019) 97:21–6. doi: 10.4174/astr.2019.97.1.21, PMID: 31297349 PMC6609415

[41] Shirota C Kawashima H Tainaka T Sumida W Yokota K Makita S. Double-balloon endoscopic retrograde cholangiography can make a reliable diagnosis and good prognosis for postoperative complications of congenital biliary dilatation. Sci Rep. (2021) 11:11052. doi: 10.1038/s41598-021-90550-7, PMID: 34040119 PMC8155203

[42] Liu F Xu X Lan M Tao B Li L Wu Q. Total versus conventional laparoscopic cyst excision and roux-en-Y hepaticojejunostomy in children with choledochal cysts: a case-control study. BMC Surg. (2020) 20:243. doi: 10.1186/s12893-020-00906-5, PMID: 33069222 PMC7568352

[43] Qu X Cui L Xu J. Laparoscopic surgery in the treatment of children with choledochal cyst. Pak J Med Sci. (2019) 35:807–11. doi: 10.12669/pjms.35.3.85, PMID: 31258599 PMC6572986

[44] Ramsey WA Huerta CT Ingle SM Gilna GP Saberi RA O'Neil CF. Outcomes of laparoscopic versus open resection of pediatric choledochal cyst. J Pediatr Surg. (2023) 58:633–8. doi: 10.1016/j.jpedsurg.2022.12.024, PMID: 36670004

[45] Xie X Li K Wang J Wang C Xiang B. Comparison of pediatric choledochal cyst excisions with open procedures, laparoscopic procedures and robot-assisted procedures: a retrospective study. Surg Endosc. (2020) 34:3223–31. doi: 10.1007/s00464-020-07560-1, PMID: 32347390

[46] Ji Y Yang K Zhang X Chen S Xu Z. Learning curve of laparoscopic Kasai portoenterostomy for biliary atresia: report of 100 cases. BMC Surg. (2018) 18:107. doi: 10.1186/s12893-018-0443-y, PMID: 30477451 PMC6260779

[47] Urushihara N Fukumoto K Yamoto M Miyake H Takahashi T Nomura A. Characteristics, management, and outcomes of congenital biliary dilatation in neonates and early infants: a 20-year, single-institution study. J Hepatobiliary Pancreat Sci. (2018) 25:544–9. doi: 10.1002/jhbp.590, PMID: 30328288

[48] Bergholz R Botden S Verweij J Tytgat S Van Gemert W Boettcher M. Evaluation of a new robotic-assisted laparoscopic surgical system for procedures in small cavities. J Robot Surg. (2020) 14:191–7. doi: 10.1007/s11701-019-00961-y, PMID: 30993523

[49] Cundy TP Shetty K Clark J Chang TP Sriskandarajah K Gattas NE. The first decade of robotic surgery in children. J Pediatr Surg. (2013) 48:858–65. doi: 10.1016/j.jpedsurg.2013.01.031, PMID: 23583146

[50] Lin Y Chen S Lin Y Zhang L Wang J Qiu X. A trans-umbilical single-site plus one robotic-assisted surgery for choledochal cyst resection in children. Front Pediatr. (2024) 12:1418991. doi: 10.3389/fped.2024.1418991, PMID: 38978841 PMC11228950

[51] Carpenter SG Grimsby G DeMasters T Katariya N Hewitt WR Moss AA. Robotic resection of choledochocele in an adult with intracorporeal hepaticojejunostomy and roux-en-Y anastomosis: encouraging progress for robotic surgical treatment of biliary disease. J Robot Surg. (2014) 8:77–80. doi: 10.1007/s11701-012-0389-5, PMID: 27637243

[52] Magouliotis DE Tasiopoulou VS Sioka E Zacharoulis D. Robotic versus laparoscopic sleeve gastrectomy for morbid obesity: a systematic review and meta-analysis. Obes Surg. (2017) 27:245–53. doi: 10.1007/s11695-016-2444-1, PMID: 27815863

[53] Chi SQ Cao GQ Li S Guo JL Zhang X Zhou Y. Outcomes in robotic versus laparoscopic-assisted choledochal cyst excision and hepaticojejunostomy in children. Surg Endosc. (2021) 35:5009–14. doi: 10.1007/s00464-020-07981-y, PMID: 32968912

[54] Ciccioli C Mazza S Sorge A Torello Viera F Mauro A Vanoli A. Diagnosis and treatment of choledochal cysts: a comprehensive review with a focus on choledochocele. Dig Dis Sci. (2025) 70:39–48. doi: 10.1007/s10620-024-08708-y, PMID: 39589463

[55] Vishwanath Reddych V Kumar A Aggarwal M Kurdia KC. Type VI choledochal cyst with gall bladder carcinoma. BMJ Case Rep. (2019) 12:e232715. doi: 10.1136/bcr-2019-232715, PMID: 31888902 PMC6936509

[56] Park DH Kim MH Lee SK Lee SS Choi JS Lee YS. Can MRCP replace the diagnostic role of ERCP for patients with choledochal cysts? Gastrointest Endosc. (2005) 62:360–6. doi: 10.1016/j.gie.2005.04.026, PMID: 16111952

[57] Vuppalanchi R Are V Telford A Young L Mouchti S Ferreira C. A composite score using quantitative magnetic resonance cholangiopancreatography predicts clinical outcomes in primary sclerosing cholangitis. JHEP Rep. (2023) 5:100834. doi: 10.1016/j.jhepr.2023.100834, PMID: 37663118 PMC10472223

[58] Goldfinger MH Ridgway GR Ferreira C Langford CR Cheng L Kazimianec A. Quantitative MRCP imaging: accuracy, repeatability, reproducibility, and cohort-derived normative ranges. J Magn Reson Imaging. (2020) 52:807–20. doi: 10.1002/jmri.27113, PMID: 32147892 PMC7496952

[59] Li H Han Z Wu H Musaev ER Lin Y Li S. Artificial intelligence in surgery: evolution, trends, and future directions. Int J Surg. (2025) 111:2101–11. doi: 10.1097/JS9.0000000000002159, PMID: 39693484

[60] Song G Jiang X Wang J Li A. Comparative clinical study of laparoscopic and open surgery in children with choledochal cysts. Saudi Med J. (2017) 38:476–81. doi: 10.15537/smj.2017.5.17667, PMID: 28439596 PMC5447207

[61] Lin Y Xu X Chen S Zhang L Wang J Qiu X. Construction of nomogram based on clinical factors for the risk prediction of postoperative complications in children with choledochal cyst. Front Pediatr. (2024) 12:1372514. doi: 10.3389/fped.2024.1372514, PMID: 39170601 PMC11337223

