# Supplementary material for: Global research trends in surgical management of choledochal cysts: a bibliometric analysis from 2000 to 2024
Source: Front Med (Lausanne). 2025 Jul 11;12:1611045. doi: 10.3389/fmed.2025.1611045 (PMC12289569; doi:10.3389/fmed.2025.1611045)

sTable 1 - The total number of articles by the top 10 authors with the proportion, the H-index, the G-index, the TC, the country, and the research direction.

| Author | Article | H-index | G-index | TC | Country |
| --- | --- | --- | --- | --- | --- |
| LI L | 38 | 17 | 26 | 718 | CHINA |
| DIAO M | 27 | 14 | 21 | 493 | CHINA |
| YAMATAKA A | 26 | 14 | 22 | 490 | JAPAN |
| LANE GJ | 23 | 11 | 18 | 344 | JAPAN |
| CHENG W | 21 | 10 | 15 | 463 | CHINA |
| DAVENPORT M | 15 | 10 | 17 | 314 | UNITED KINGDOM |
| KOGA H | 14 | 10 | 11 | 264 | JAPAN |
| TAINAKA T | 13 | 9 | 10 | 245 | JAPAN |
| XIANG B | 13 | 9 | 10 | 276 | CHINA |
| IEIRI S | 12 | 8 | 11 | 122 | JAPAN |

^a^ Abbreviations: TC = Total Citation

sTable 2 - The numbers and country of the top 10 sources with the most publications.

| Source | Article | Country | IF | JCR |
| --- | --- | --- | --- | --- |
| JOURNAL OF PEDIATRIC SURGERY | 99 | UNITED STATES | 2.4 | Q1 |
| PEDIATRIC SURGERY INTERNATIONAL | 87 | GERMANY | 1.5 | Q2 |
| JOURNAL OF LAPAROENDOSCOPIC & ADVANCED SURGICAL TECHNIQUES | 39 | UNITED STATES | 1.1 | Q2 |
| SURGICAL ENDOSCOPY AND OTHER INTERVENTIONAL TECHNIQUES | 36 | UNITED STATES | 2.4 | Q1 |
| HEPATO-GASTROENTEROLOGY | 24 | ITALY | / | / |
| INTERNATIONAL JOURNAL OF SURGERY CASE REPORTS | 22 | UNITED KINGDOM | 0.6 | Q3 |
| WORLD JOURNAL OF SURGERY | 22 | UNITED STATES | 2.3 | Q1 |
| EUROPEAN JOURNAL OF PEDIATRIC SURGERY | 20 | GERMANY | 1.5 | Q2 |
| FRONTIERS IN PEDIATRICS | 20 | SWITZERLAND | 2.1 | Q2 |
| JOURNAL OF PEDIATRIC SURGERY CASE REPORTS | 20 | UNITED STATES | 0.2 | Q4 |

^a^ Abbreviations: IF = Impact Factor; JCR = Journal Citation Reports quartile

sFigure1. Study Screening Flowchart


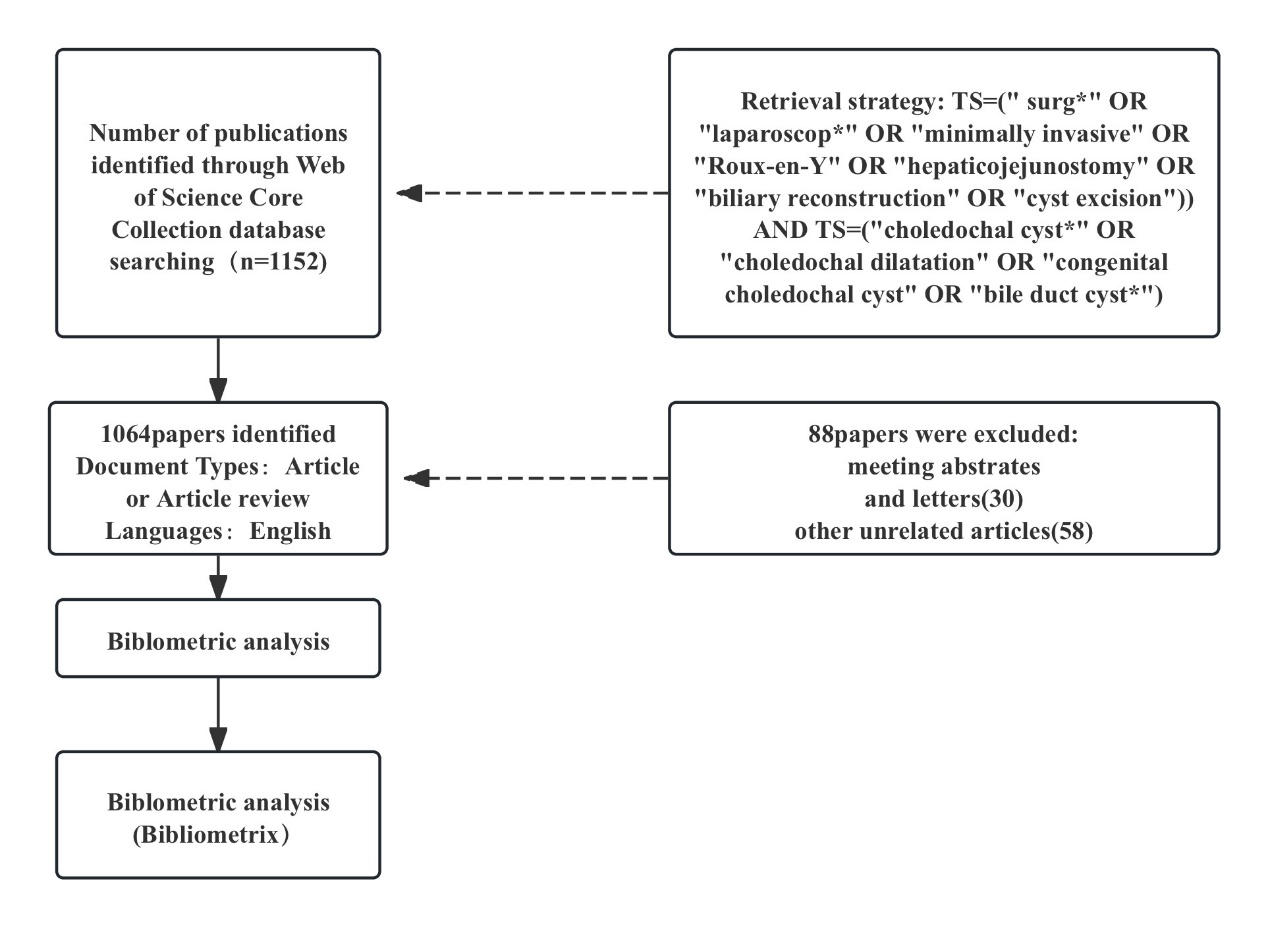


sFigure2. Collaborative relationships among researchers


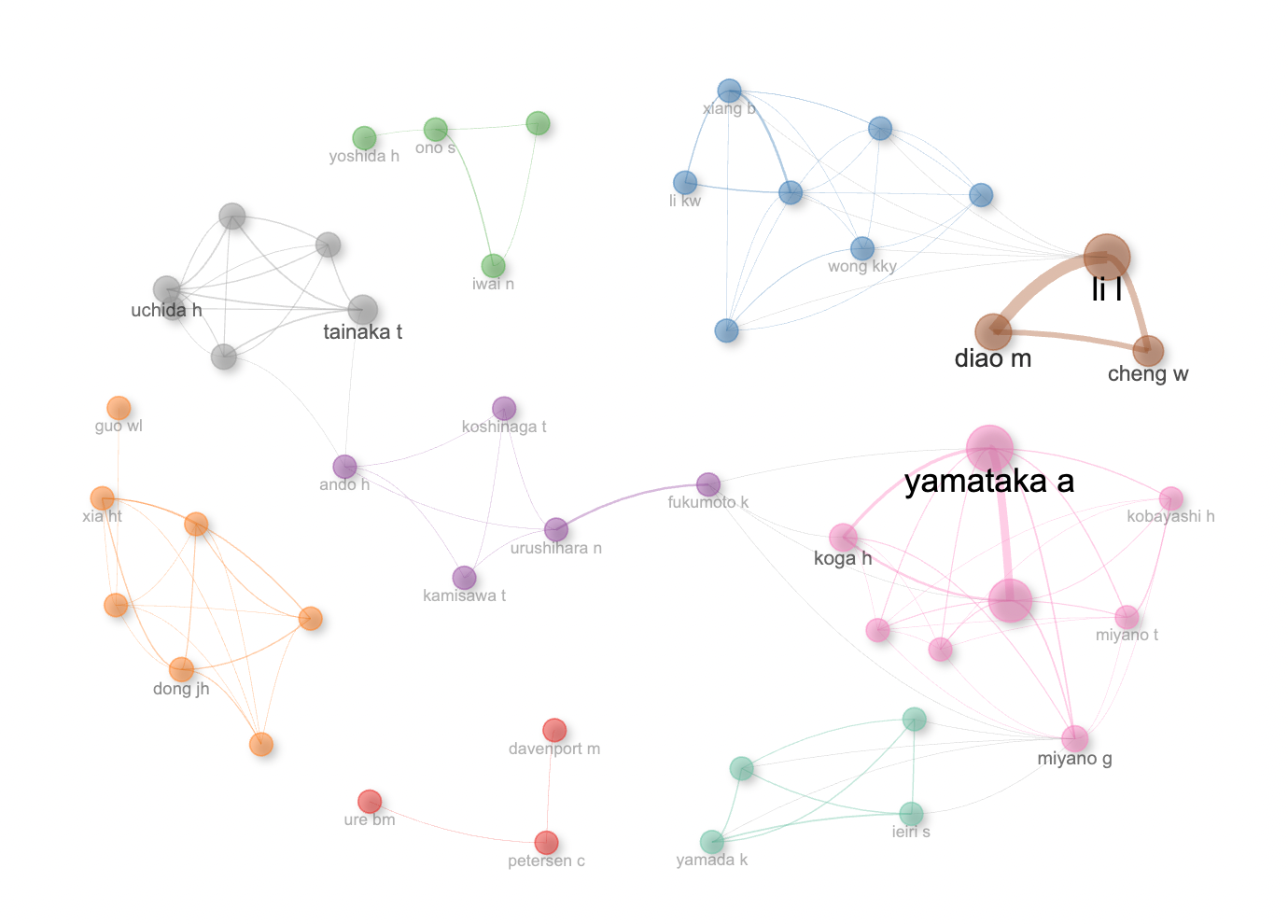


sFigure3. The publications trend among the top organizations from 2000 to 2024.


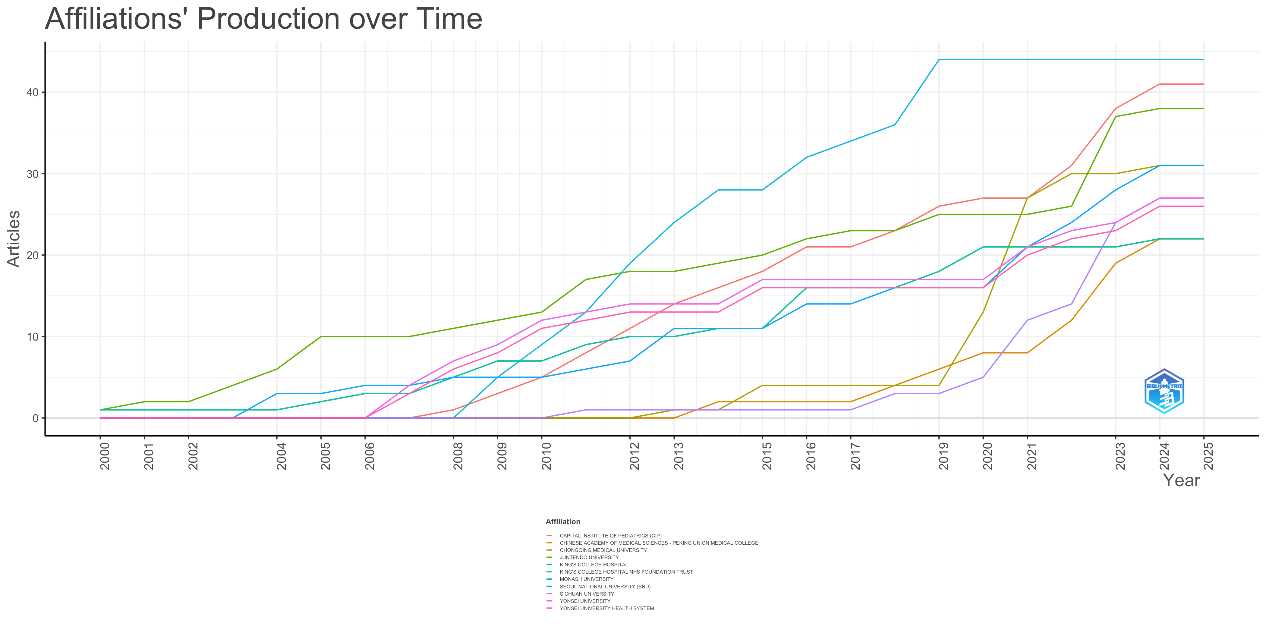


sFigure4. Top 10 global organizations focusing on surgery for CCs.


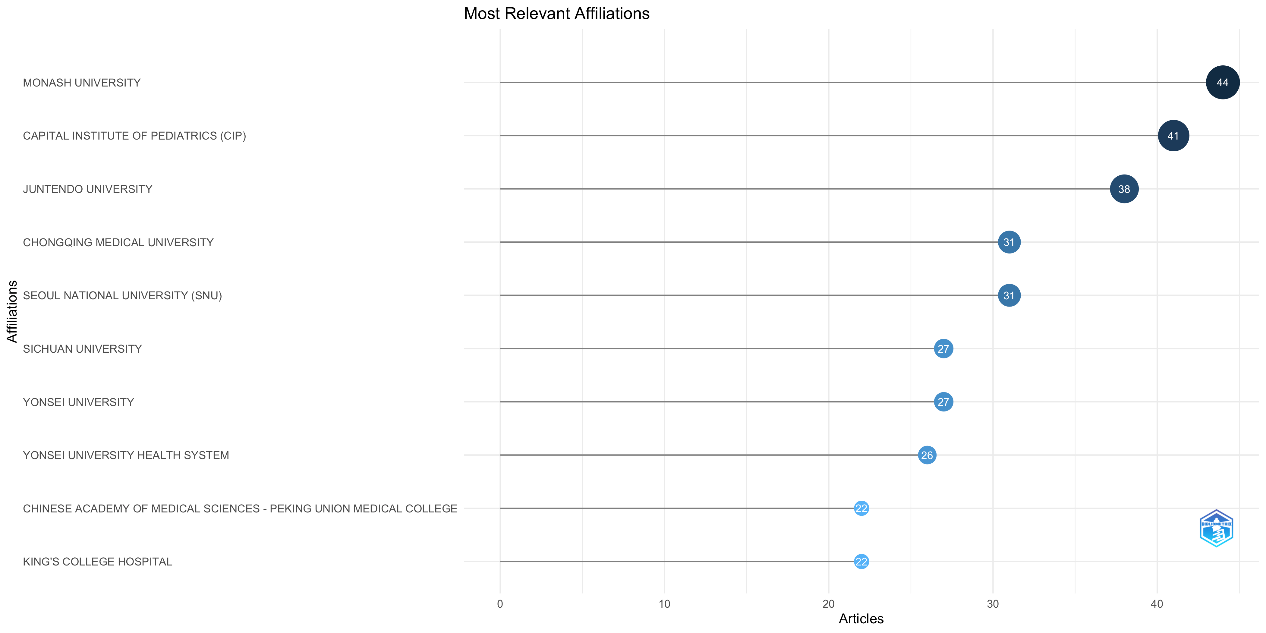


sFigure5. Network visualization between the top countries, organizations and keywords.


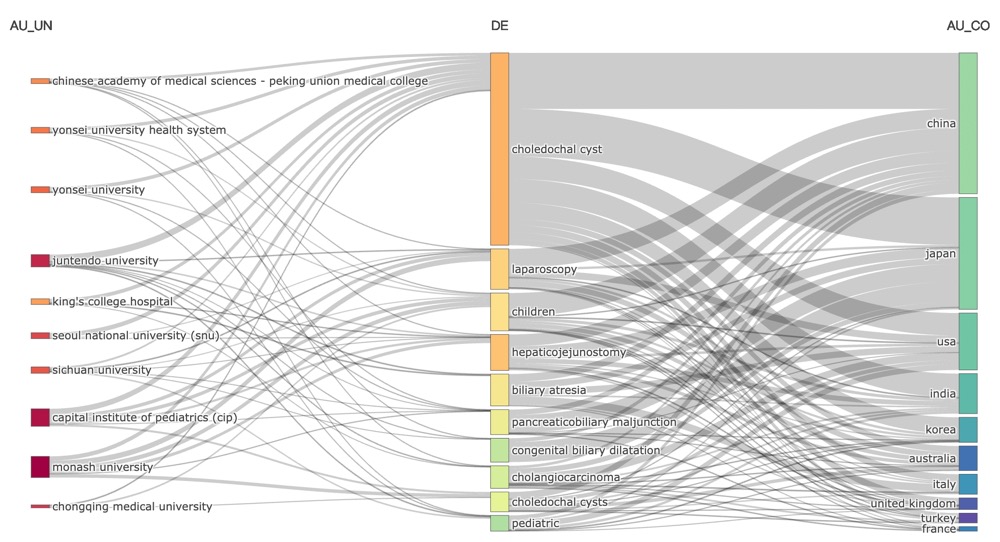

Supplement: Supplementary file 1 [file Data_Sheet_1.docx]
